# Assessing experience in the deliberate practice of running using a fuzzy decision-support system

**DOI:** 10.1371/journal.pone.0183389

**Published:** 2017-08-17

**Authors:** Maria Isabel Roveri, Edison de Jesus Manoel, Andrea Naomi Onodera, Neli R. S. Ortega, Vitor Daniel Tessutti, Emerson Vilela, Nelson Evêncio, Isabel C. N. Sacco

**Affiliations:** 1 University of São Paulo, School of Medicine, Physical Therapy, Speech and Occupational Therapy dept., São Paulo, SP, Brazil; 2 Study Group in Action Development and Motor Intervention, School of Physical Education and Sport, University of São Paulo, São Paulo, SP, Brazil; 3 Dass Nordeste Calçados e Artigos Esportivos Inc, Ivoti, Rio Grande do Sul, Brazil; 4 School Of Medicine, University Of São Paulo, São Paulo, SP, Brazil; 5 Centro Universitário Fieo. Osasco, São Paulo, Brazil; Southwest University, CHINA

## Abstract

The judgement of skill experience and its levels is ambiguous though it is crucial for decision-making in sport sciences studies. We developed a fuzzy decision support system to classify experience of non-elite distance runners. Two Mamdani subsystems were developed based on expert running coaches’ knowledge. In the first subsystem, the linguistic variables of training frequency and volume were combined and the output defined the quality of running practice. The second subsystem yielded the level of running experience from the combination of the first subsystem output with the number of competitions and practice time. The model results were highly consistent with the judgment of three expert running coaches (r>0.88, *p*<0.001) and also with five other expert running coaches (r>0.86, *p*<0.001). From the expert’s knowledge and the fuzzy model, running experience is beyond the so-called "10-year rule" and depends not only on practice time, but on the quality of practice (training volume and frequency) and participation in competitions. The fuzzy rule-based model was very reliable, valid, deals with the marked ambiguities inherent in the judgment of experience and has potential applications in research, sports training, and clinical settings.

## Introduction

“Experience” is an ambiguous term. The boundary between what is experience and what is not is blurry, and even more so when levels of experience are to be defined. Yet such definition is crucial in many studies in sports sciences. To evaluate the effect of training regimes properly, the status of athletes in terms of experience must be definitively assessed. Novice and skilled, or novice and highly trained, or expert and non-expert are all terms often used to categorize athletes, although they tend to be very study-specific, where the definitions of the terms rarely apply beyond the study in which they are used [1][2][3]. That is, the criteria for defining an experienced athlete in one study may be quite different from those used in other studies [4][5][6][7]. There is little we can do about this because, among other reasons, such ambiguity is inherent to the nature of the experience phenomenon. We therefore need an alternative to the traditional bivalent logic that defines a thing as either this or that.

We refer to the fuzzy logic proposed by Zadeh [8] for dealing with the ambiguity inherent in many biological, psychological, and sociological phenomenon. Fuzzy logic supports efforts to deal with elements that do not fall into one or another class in a dichotomous way, but are rather a matter of degree [9]. The application of fuzzy logic has been successful in the realm of diagnosis [10][11][12][13][14] because a combination of various criteria may deliver the best possible judgment about the patient’s disease [15]. We think that the definition of experience in running has features similar to those in clinical diagnosis, as both involve dealing with fuzzy sets.

In the present paper, we set out to develop a computer-based system using fuzzy logic to classify experience in recreational runners. Recreational running is a growing social phenomena around the world [16][17], and such a level of involvement warranted an interest from the fields of biomechanics and sports. The definition of a runner’s experience is important for the decision-making processes of choosing appropriate footwear and planning training sessions or rehabilitation procedures. The classification of experience, however, has been arbitrary and prone to wide variability.

Most of the literature on the subject of experience deals with cognitive skills and it has come to represent a paradigm for defining and understanding experience and expertise in sports [18]. The so-called 10-year rule is widely accepted for the definition of an expert [19]. However, this rule became confused with time exposed to a given activity and the amount of practice dedicated exclusively to excelling in that activity. But, the steady and long involvement with some activity is not sufficient for one to become an expert. Ericsson and colleagues [20][21] emphasized that, together with the steady involvement with an activity, one must have deliberate practice, a practice that provides effective acquisition of specific skills, to become an expert. Studies in sports have considered the expertise paradigm and analysed differences among athletes (experienced and novice, elite and non-elite, etc.) in the development of expertise [22][23][24][25].

Deliberate practice has been recognized as central to the development of expertise ever since the seminal paper of [20]. A runner’s experience could then be examined in terms of the amount of deliberate practice, but years of deliberate practice may not be enough to classify the experience of a runner [6]. This is because the structure of the deliberate practice matters for defining experience and the development of expertise [26]. Experience in running is also not necessarily referenced by performance level. There are many recreational runners all over the world who cannot match Olympic standards, as Young & Salmela [6] defined, but who are still able to perform quite well in distance running, such as finishing a marathon in less than 3:30 or even 3:00 hours, or a 10k race in less than 35 minutes. The pace required to achieve those times are not met occasionally for a non-runner, they entail a runner’s involvement in serious and regular practice and training, which may be different from the involvement of a professional runner.

The structure of a deliberate running practice can be oriented by the amount of practice (weeks, months, or years); the frequency, volume, and intensity of running; being coached and the time spent with a coach; the kind and amount of other practices such as weight and technique training [6]; and the participation in competitions. Baker, Horton & Robertson-Wilson [22] argued that taking part in competitions could enhance the experience and function of gaining expertise in sport because it could be considered as a special practice session that cannot be reproduced during common deliberate practice. Overall, these elements can be quantified and reported by the runner, and most coaches use such information to decide on the degree of a runner’s capability, and therefore to plan the training schedules over various cycles.

In this context, we aimed to develop a fuzzy decision-support system using an expert knowledge system for classifying the experience of non-professional long-distance runners.

## Methods

### Development of the rule-based fuzzy model

Fuzzy rule-based models are systems whose variables are described by fuzzy sets rather than crisp numbers. They are based on the concept of fuzzy partitioning of the information and on the Approximate Reasoning, which provides a framework for reasoning with uncertain information. Fuzzy linguistic model could be defined as a particular expert system, composed basically by a knowledge base and an inference engine, both are based on the information provided by the experts, in our case the running coaches. The greater advantage of a fuzzy system is to allow considering the identification uncertainty present in any evaluate process in the built model.

The development of the rule-based fuzzy model involved the following steps: (1) defining the model goal–experience in running by long-distance recreational runners, based on consensus between experts about the most relevant input variables (2) defining the linguistic variables as model inputs, (3) defining the output variables of the fuzzy model, (4) building fuzzy if-then rules with the input variables and logic connectives, which consists in a set of conditional fuzzy propositions, and (5) defining a defuzzification method, which translates fuzzy outputs back to crisp values, in case a numeric classical output is needed [14].

The if-then fuzzy rule model can be understood as a mathematical mapping of a fuzzy input space into fuzzy output space. A fuzzy rule system is analogous to a mathematical function, as a fuzzy relation on the Cartesian product space, able to describe both linear and non-linear phenomenon. The inference method used was the Mamdani method, which uses minimum for the conjunction operator and maximum for the disjunction operator.

The if-then fuzzy rules are structures widely applied in several approaches of fuzzy sets theory and they provide a formal way to represent information from experiences and empirical associations. The IF-part of the rule describes a condition, or assumptions, that can be partially satisfied, and the THEN-part describes a conclusion, or an action, that can be found when the conditions have been hold. Assuming that the experience-phenomenon is well represented in the model, a set of rules is built from the combination of the input variables selected. The if-then fuzzy rules were used to evaluate the quality of practice and the running experience both being based on the knowledge of experts.

Defuzzification is a procedure that allows the interpretation about the possibility of the fuzzy output distribution in a quantitative way. Center of Area method, the chosen defuzzification method, considers the entire possibility distribution to calculate the defuzzified value, which usually provides the most representative crisp number in the variable domain [13].

The fuzzy models were implemented in MATLAB v.15 (available online) using the fuzzy logic toolbox [27]. The study was approved by the “Ethics Committee of the School of Medicine of the University of Sao Paulo” (Ethical Application CEP-FMUSP: protocol no. 030/15). All written informed consents were obtained from all runners and participants involved, which followed the principles of the Declaration of Helsinki.

### Definition of the model’s structure, system’s input and output variables

The structure of the model was defined in terms of the experience and knowledge of three expert running coaches and one expert on fuzzy modelling. The long-term running coaches had at least 10 years of coaching experience in addition to the experience they had as runners.

*Expert 1*: male, degree in Physical Education (teaching degree), graduation in Sport training, runner and triathlete, and running coach since 2001, coaching mainly marathon athletes in Brazil and around the world. He has run the São Silvestre, Rio de Janeiro marathon, and Ironman 70.3.

*Expert 2*: male, degree in Sport Studies & Training (bachelor degree), graduation in Sport training, masters in Rehabilitation Science, runner for more than 30 years, running coach since 2000, coaching long distance national athletes. He has experience in triathlon runs and mountain running (40 and 100km).

*Expert 3*: male, degree in Physical Education (teaching degree), International Association of Athletics Federations accredited athletics coach Level II, middle- and long-distance runner for 21 years, chair of the Running Coaches Association of São Paulo since 2009, running coach since 1994, coaching recreational and professional runners of half and long distance.

The coaches participated in five meetings chaired by the researchers and the fuzzy modeling expert. These meetings concerned what is experience in running and the factors, independent from the performance level, that affect experience development and acquisition. The meetings developed linguistic variables that were translated into the fuzzy rule-based model to classify running experience. Initially, two linguistic variables were identified and considered in combination as qualities of deliberate practice: training frequency (number of running sessions per week) and training volume (distance in km run per week). Then the experts raised two more linguistic variables to define experience: practice time (years of running practice) and number of competitions (quantity of street competitions in which the runner took part).

**Fig 1:**
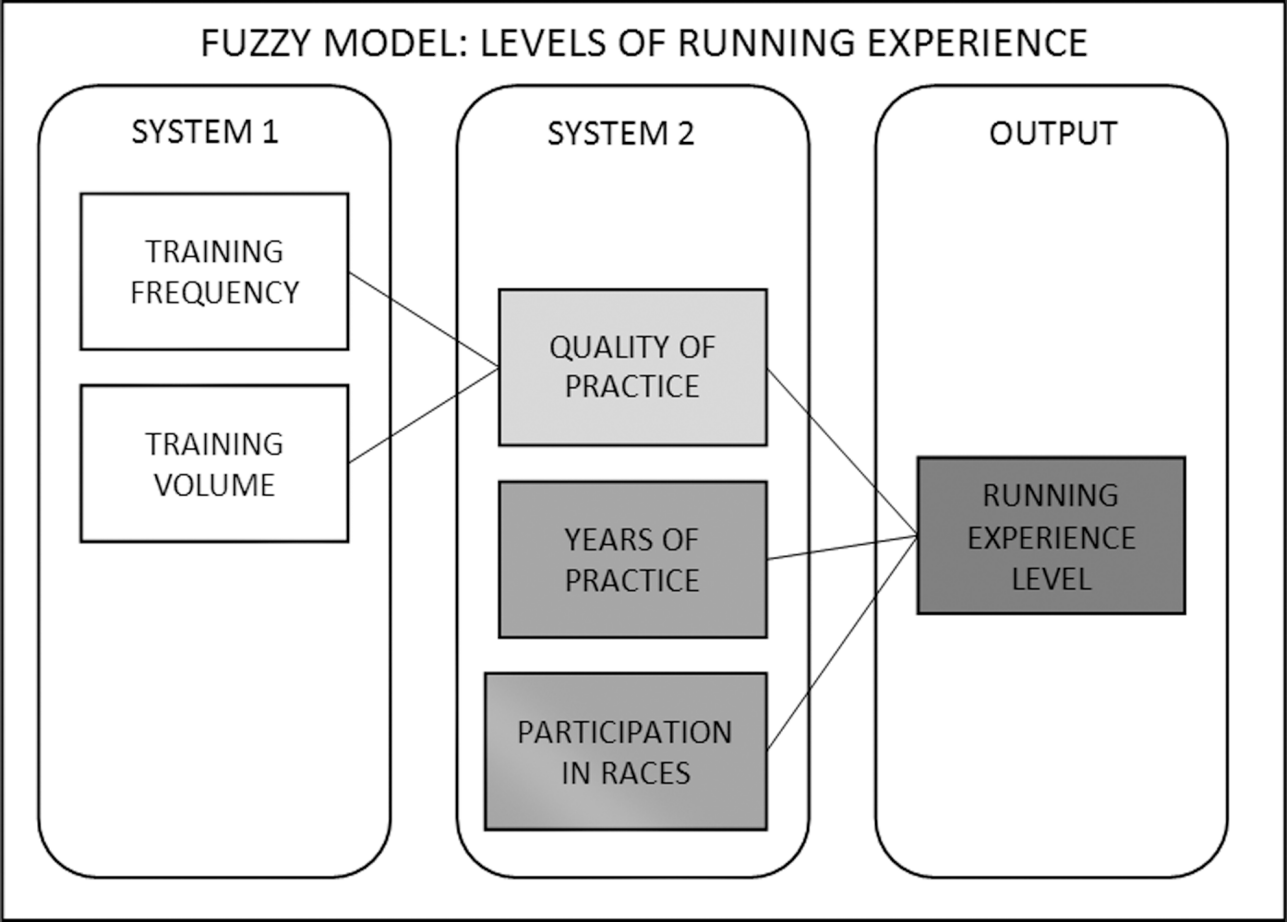
Representation of the fuzzy model with two subsystems and their respective sets, and the output set of the model.

The fuzzy rule-based model to classify running experience consisted of two subsystems that combined the linguistic variables discussed by the experts (Fig 1). Subsystem 1 entailed training frequency and training volume. Its output (quality of deliberate practice) was used as input for subsystem 2, which combined quality of practice with practice time and number of competitions; its output was the level of running experience.

The input variables in subsystem 1 were fuzzified using the following linguistic terms, based on the coaches experts opinion.

- Training frequency: (1) too low, (2) low, (3) medium, or (4) high
- Training volume: (1) too low, (2) low, (3) medium, or (4) high

Accordingly, the input variables in subsystem 2 were fuzzified using the following linguistic terms.

- Quality of practice: (1) very bad, (2) bad, (3) medium, (4) good, or (5) very good
- Number of competitions: (1) few, (2) medium, or (3) many
- Practice time: (1) very short, (2) short, (3) moderate, or (4) long

**Fig 2:**
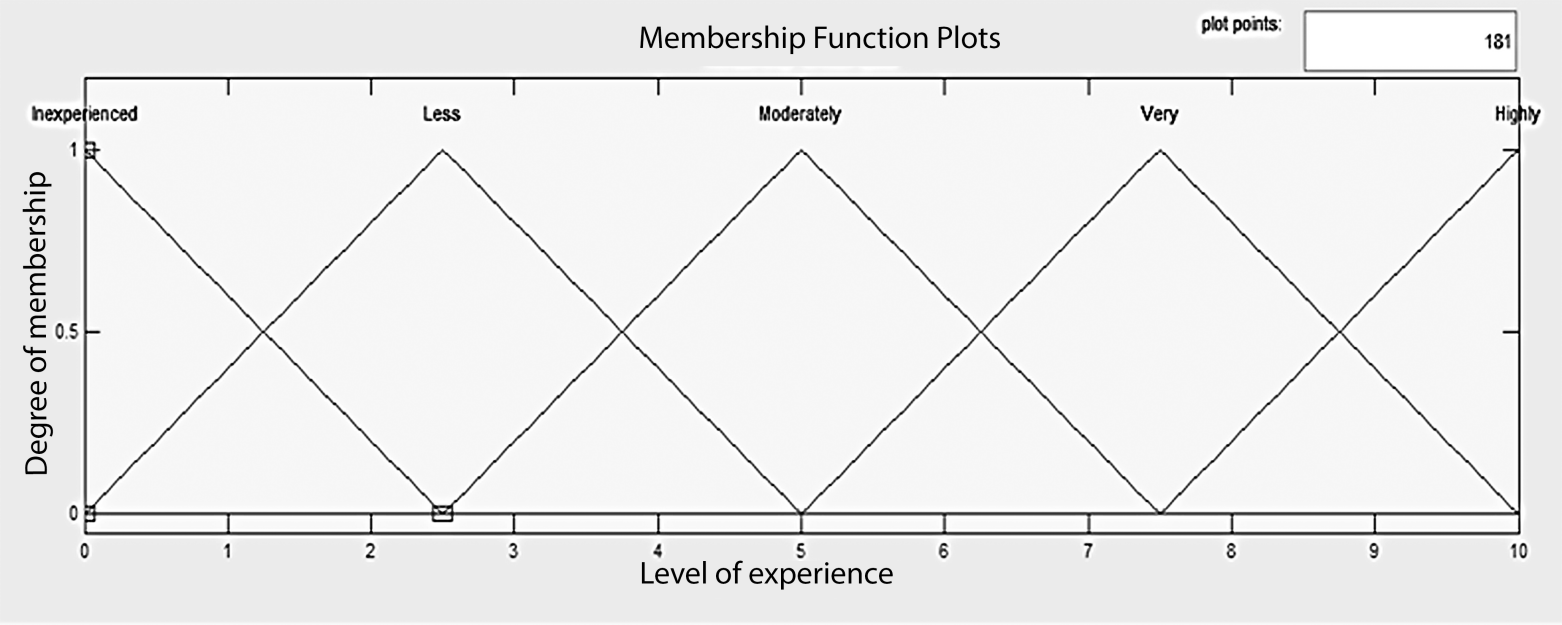
Output sets of the final fuzzy model: Levels of running experience.

The output fuzzy sets for system 2 included four running experience levels: (I) inexperienced, (II) less experienced, (III) moderately experienced, (IV) very experienced, and (V) highly experienced. The level of running experience was represented as a score in the interval [0,10], where 0 means “no experience” and 10 means “great experience”. Since there was no reason to assume non-linear conditions in the classification of experience on the domain considered, triangular membership functions were used. The support of the fuzzy sets was partitioned in a homogeneous way, covering all variable domains and avoiding inconsistences in the system (Fig 2).

Based on the fuzzy input sets, linguistic rules were elaborated using a combinatory analysis and this ensures that all possible circumstances were considered. For each rule experts pointed what was the proper output, by an empirical association. This led to a different number of rules for each subsystem: 16 rules for subsystem 1 and 60 rules for subsystem 2. The experts determined the consequence of each fuzzy rule, which was elaborated in the form of the following example. For subsystem 1: “IF training frequency is *too low* AND training volume is *too low* THEN practice quality is *too bad*.”

For subsystem 2: “IF practice quality is *too bad* AND number of competitions is *few* AND practice time is *too short* THEN experience level is *inexperienced*.”

Tables 1 and 2 present the rules used in the subsystems 1 and 2, respectively.

**Table 1:** Rules used in the subsystem 1 –quality of practice.

Rules—subsystem 1	Training Volume	Training Frequency	Quality of Practice
**1**	Too low	Too low	Very bad
**2**	Too low	Low	Very bad
**3**	Too low	Medium	Very bad
**4**	Too low	High	Very bad
**5**	Low	Too low	Bad
**6**	Low	Low	Bad
**7**	Low	Medium	Bad
**8**	Low	High	Bad
**9**	Medium	Too low	Medium
**10**	Medium	Low	Medium
**11**	Medium	Medium	Medium
**12**	Medium	High	Medium
**13**	High	Too low	Bad
**14**	High	Low	Good
**15**	High	Medium	Good
**16**	High	High	Very good

**Table 2:** Rules used in the subsystem 2 –experience level.

Rules—subsystem 2	Number of Competitions	Quality of Practice	Practice Time	Experience
**1**	Few	Very bad	Very short	Inexperienced
**2**	Few	Very bad	Short	Inexperienced
**3**	Few	Very bad	Moderate	Inexperienced
**4**	Few	Very bad	Long	Less experienced
**5**	Few	Bad	Very short	Inexperienced
**6**	Few	Bad	Short	Inexperienced
**7**	Few	Bad	Moderate	Less experienced
**8**	Few	Bad	Long	Less experienced
**9**	Few	Medium	Very short	Less experienced
**10**	Few	Medium	Short	Moderately experienced
**11**	Few	Medium	Moderate	Moderately experienced
**12**	Few	Medium	Long	Moderately experienced
**13**	Few	Good	Very short	Moderately experienced
**14**	Few	Good	Short	Moderately experienced
**15**	Few	Good	Moderate	Very experienced
**16**	Few	Good	Long	Very experienced
**17**	Few	Good	Very short	Moderately experienced
**18**	Few	Good	Short	Very experienced
**19**	Few	Good	Moderate	Very experienced
**20**	Few	Good	Long	Highly experienced
**21**	Medium	Very bad	Very short	Inexperienced
**22**	Medium	Very bad	Short	Inexperienced
**23**	Medium	Very bad	Moderate	Less experienced
**24**	Medium	Very bad	Long	Less experienced
**25**	Medium	Bad	Very short	Less experienced
**26**	Medium	Bad	Short	Less experienced
**27**	Medium	Bad	Moderate	Less experienced
**28**	Medium	Bad	Long	Less experienced
**29**	Medium	Medium	Very short	Moderately experienced
**30**	Medium	Medium	Short	Moderately experienced
**31**	Medium	Medium	Moderate	Moderately experienced
**32**	Medium	Medium	Long	Very experienced
**33**	Medium	Good	Very short	Moderately experienced
**34**	Medium	Good	Short	Very experienced
**35**	Medium	Good	Moderate	Very experienced
**36**	Medium	Good	Long	Highly experienced
**37**	Medium	Very good	Very short	Moderately experienced
**38**	Medium	Very good	Short	Very experienced
**39**	Medium	Very good	Moderate	Highly experienced
**40**	Medium	Very good	Long	Highly experienced
**41**	Many	Very bad	Very short	Inexperienced
**42**	Many	Very bad	Short	Inexperienced
**43**	Many	Very bad	Moderate	Less experienced
**44**	Many	Very bad	Long	Less experienced
**45**	Many	Bad	Very short	Less experienced
**46**	Many	Bad	Short	Inexperienced
**47**	Many	Bad	Moderate	Moderately experienced
**48**	Many	Bad	Long	Moderately experienced
**49**	Many	Medium	Very short	Less experienced
**50**	Many	Medium	Short	Moderately experienced
**51**	Many	Medium	Moderate	Moderately experienced
**52**	Many	Medium	Long	Very experienced
**53**	Many	Good	Very short	Moderately experienced
**54**	Many	Good	Short	Very experienced
**55**	Many	Good	Moderate	Highly experienced
**56**	Many	Good	Long	Highly experienced
**57**	Many	Very good	Very short	Very experienced
**58**	Many	Very good	Short	Very experienced
**59**	Many	Very good	Moderate	Highly experienced
**60**	Many	Very good	Long	Highly experienced

**Fig 3:**
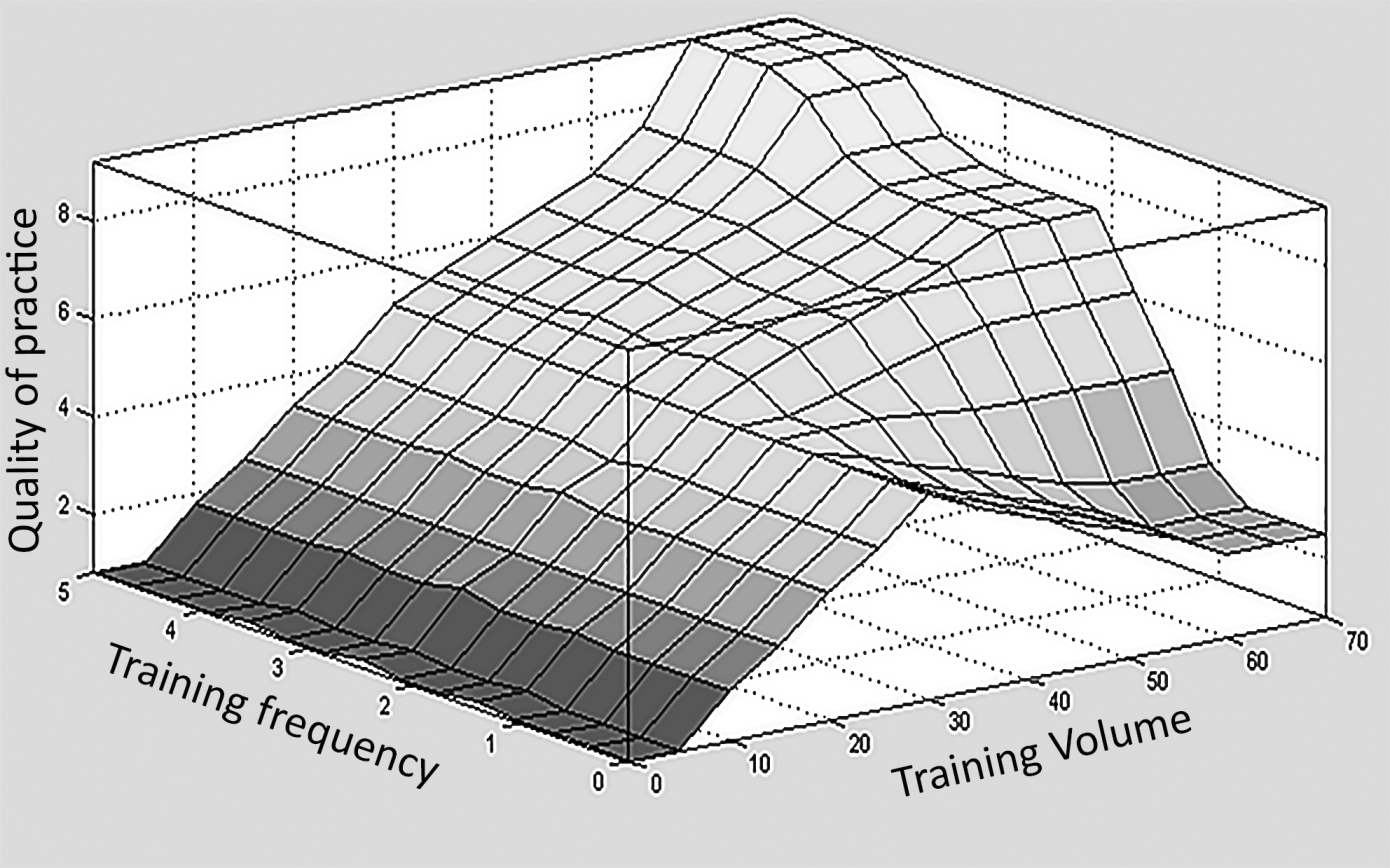
Surface graph representation of the quality of practice in Relation to training frequency and training volume.

**Fig 4:**
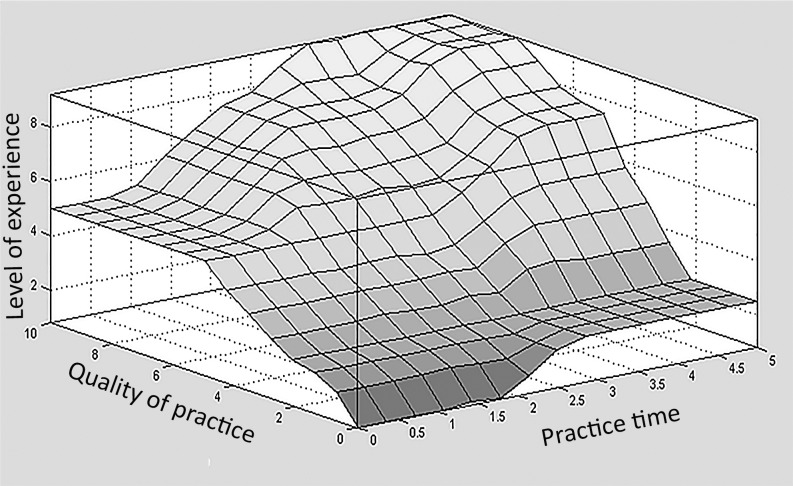
Surface graph representation of the levels of running experience in relation to quality of practice and practice time.

Figs 3 and 4 present the surface graphs. These graphs give a tri-dimensional representation of the relationship between two input variables of the model and the corresponding output variable, similar to a mathematical function in which input variables were mapped into output ones. From this analysis, it was possible to judge the homogeneity of the distribution and the distribution’s relationship with the output variable. The surface graphs represent aspects of the mathematical behavior of the rule-based model. The vertical axis corresponds to the output variable of interest. In Fig 3, the surface graph presents the quality of practice related to training frequency and training volume.

The experience level is presented in Fig 4. Experience level is related to quality of practice and practice time. The behavior of the variables indicates that practice time becomes important after 1.5 years of training. Still, it is the quality of practice that principally influences the experience level up to score 6.

### Refining and analyzing model performance

To refine subsystem 1, we used a dataset consisting of 30 hypothetical cases of training frequency and training volume. These cases were evaluated independently by the three expert running coaches and the results were compared by the output of subsystem 1 (quality of practice).

To refine the subsystem 2, a new dataset composed of 62 hypothetical cases was created. These cases involved quality of practice, number of competitions, and practice time. This new dataset was also evaluated by the three expert running coaches and their results were compared with the output of subsystem 2.

The experts evaluated each case in both datasets (two hypothetical) giving their judgments on an analogical visual scale (0 to 10), in which they had to make a mark in a continuum that went from inexperienced to experienced.

The next step was to analyze the performance of the fuzzy model, comparing its results for the real data set to those of the expert judgments on the same dataset. The real dataset was composed of 100 adult non-professional runners who gave their informed consent to participate; they were interviewed about their training volume (25.4±16.6 km/week), frequency (3±1 times/week), practice time (5.0±5.2 years), and number of competitions (27±63 races). The experts evaluated each case in the real dataset the same way they performed in the hypothetical ones. Then, Pearson’s correlation product was calculated between the experts judgments using the analog scale in the real dataset (n = 100) and the score given by the fuzzy rule-based model subsystems 1 and 2 (normality confirmed by Shapiro Wilk test).

Since there is no gold standard that identifies the level of experience in running, another 5 running experts (numbered 4 to 8) judged the experience level of runners from the real dataset (n = 100); they considered the following variables: training volume and frequency, practice time, and number of competitions. They also had access to the age and sex of the runner, whether each had a coach or not, and each personal best time in 5km or 10km. Using this information, they had to classify each runner in one of four category levels of experience: experienced, moderately experienced, less experienced, and inexperienced. After one week, they were again asked to classify the same runners from the dataset, giving their judgments on the analogical visual scale. We also correlated the evaluations performed by these five new experts and the model output by Pearson’s correlation (normality confirmed by Shapiro Wilk test). These new experts were running coaches who also had experience as runners.

*Expert 4*: male, degree in Physical Education (bachelor degree), middle- and long-distance runner (5k, 10k, and 20k), running coach for 15 years, coaching adult and elderly street runners.

*Expert 5*: female, degree in Physical Education (bachelor degree), graduation in Exercise physiology, long-distance runner for 10 years, running coach for 11 years, coaching long-distance street runners, and ultramarathon athletes.

*Expert 6*: male, degree in Physical Education (teaching degree), graduation in Exercise physiology, long-distance runner for 16 years, running coach for 13 years, coaching 120 recreational and professional runners in a regular basis.

*Expert 7*: male, degree in Physical Education (teaching degree), graduation in Exercise physiology, Biomechanics and Marketing MBA, long-distance runner for 20 years, running coach for 14 years. He has run 15 marathons and 4 ultramarathons, twice the Comrades.

*Expert 8*: male, degree in Physical Education and Athletics Training (teaching degree), Master and PhD degree in Physical Education, long-distance runner for 35 years, running coach for 4 years for professional athletes.

To evaluate the performance of the experts’ judgment in the real database (n = 100), we also performed the kappa coefficient of agreement, which quantified the correlation among the categorical judgments made by the second group of 5 experts. We considered the categories poor agreement (0–0.19), fair (0.20–0.39), moderate (0.40–0.59), substantial (0.60–0.79), almost perfect (0.80–1.00) [28]. The numerical scores from the model were translated into the same categorical variables used by the experts to judge the level of experience, in order to perform the Kappa analysis. This translation was performed by the coaches based on their expert opinions. Fuzzy scores from 7 to 10 were categorized as *experienced*, scores smaller than 7 and greater or equal to 5 were considered *moderately experienced*, scores less than 5 and greater or equal to 2.5 were considered *less experienced*, and scores smaller than 2.5 were considered *inexperienced*.

## Results

### Comparison with the first group of experts (1, 2, and 3): Model reliability

Tables 3 and 4 present the correlations between experts 1, 2, and 3 and fuzzy subsystems 1 (quality of practice) and 2 (level of experience). Correlation was statistically significant with *r* values greater than 0.88 (*p* < 0.001).

**Table 3:** Pearson’s correlation (r) between the three experts scores and the output of the fuzzy subsystem 1 –quality of practice in the real dataset (n = 100).

	Expert 1	Expert 2	Expert 3	Mean
**Expert 1**	-	0.849	0.693	-
**Expert 2**	0.849	-	0.792	-
**Expert 3**	0.693	0.792	-	-
**Model**	0.899	0.979	0.767	0.978

**Table 4:** Pearson’s correlation (r) between the three experts scores and the output of the fuzzy subsystem 2 –level of running experience in the real dataset (n = 100).

	Expert 1	Expert 2	Expert 3	Mean
**Expert 1**	-	0.922	0.891	-
**Expert 2**	0.922	-	0.907	-
**Expert 3**	0.891	0.907	-	-
**Model**	0.878	0.916	0.902	0.928

### Comparison with the second group of experts (4 to 8): Model validity

Table 5 presents the correlations for the real dataset between experts 4, 5, 6, 7, and 8 and the fuzzy model output of level of experience. Correlation was again statistically significant with *r* values greater than 0.75 (*p* < 0.001).

**Table 5:** Pearson’s correlation (r) between the five new experts scores and the output of the fuzzy subsystem 2 –level of running experience in the real dataset (n = 100).

	Model	Expert 4	Expert 5	Expert 6	Expert 7	Expert 8
**Model**	-	0.950	0.858	0.863	0.872	0.860
**Expert 4**	0.950	-	0.856	0.885	0.866	0.842
**Expert 5**	0.858	0.856	-	0.851	0.775	0.745
**Expert 6**	0.863	0.885	0.851	-	0.827	0.805
**Expert 7**	0.872	0.866	0.775	0.827	-	0.896
**Expert 8**	0.860	0.842	0.745	0.805	0.896	-

Table 6 presents the agreement between expert judgment in categories and the categories translated from the numerical output of the model. The kappa coefficient indicated an overall fair agreement between the experts and the model (kappa = 0.337). Nevertheless, the agreement was substantial for the category *experienced* (kappa = 0.650). The category for which experts and the model seemed to be poor agreement was *less experienced* (kappa = 0.161).

**Table 6:** Kappa coefficients of agreement among the five experts in each category classification and the model’s categories, and the general agreement of all classifications of running experience in the real dataset (n = 100).

	Experienced	Moderately Experienced	Less Experienced	Inexperienced	General
**kappa**	0.650	0.202	0.161	0.365	0.337
**p-value**	< 0.001	< 0.001	0.001	< 0.001	< 0.001

## Discussion

Because the definition of experience in recreational long-distance runners is still arbitrary and prone to wide variance among authors, we developed a computer-based system using fuzzy logic to classify this phenomenon. Defining a runner’s experience is important for the decision-making processes that concern training plans or a rehabilitation process. The fuzzy rule-based model developed was very reliable, valid, and seemed to be appropriate because it not only provided an objective and systematic way of classifying experience, but also dealt with the marked ambiguities inherent in the judgment process. Currently and routinely, we count on the tacit knowledge of a running coach to classify a runner’s experience level. Each coach has many forms of assessment and his or her own experience to recognize a practitioner's experience. To the best of our knowledge, there is no mathematical model or quantitative tool that classifies the level of experience in running. The mathematical fuzzy model developed, however, systematized the knowledge of running experts, turning it into linguistic variables, which in turn were transformed into fuzzy sets. These enabled the automatized classification of a recreational runner’s experience level for broad and public usage. The fuzzy rule-based model yielded good results in terms of reliability and validity.

Previous studies that compared different groups of runners usually categorized them as elite and non-elite based on performance level, whether the runners took part in competitions or runners’ clubs, and whether they did so regularly. Such differentiations are gross, based on very disparate features that say little about one’s experience in running. When expert knowledge was assessed via interviews with experienced coaches, we gained higher-quality information about particular features of performance that contributes to the understanding of sport performance’s constraints [29]. Nevertheless, when expert running coaches were asked to declare what constitutes an experienced runner, there was an element of vagueness in their judgment that was similar to that observed in expert judgments in the medical sciences [11][13].

We started out by asking expert running coaches how they identify experience in running. They agreed with the literature in that they valued particular aspects of deliberate practice such as training frequency and volume [6] and number of competitions [30], rather than only the overall duration of practice.

The reliability of the model developed can be judged by the results of the first analysis with the first group of experts. This group participated in meetings that identified and defined the linguistic variables that later underwent the fuzzification process. Using hypothetical and real datasets, the high correlations found between their judgments and those emerging from the model indicated that the model represented expert knowledge about running experience well. The expert running coaches did not have access to the scores generated by the model when they were asked to judge the same datasets. The fact that correlation was high corroborates the reliability of the model in the sense that the model somehow reflected the expert knowledge inserted in it.

At this stage, the fuzzy model presented an interesting feature of the complexity involved in the process of gaining experience. The surface graphs indicated that a long practice time is not enough to predict good scores on experience level; the quality of practice must also be taken into account (Fig 4). The fuzzy set “practice time” that was defined by experts considered more than 4 years of running training as a long-time practice; after including other linguistic variables in the model, we would have ranked such a runner at the highest level of experience: “highly experienced.” The tridimensional relationship between duration of practice time, quality of practice, and experience level agrees with what the literature has indicated, that particular features of deliberate practice seem to be more valuable to expert performance than only the amount of time spent in practice [30][31][32][33].

The validity of the model was tested by comparing model output to the judgments of a separate group of experts. The results were encouraging because the correlations found between each expert and the model (see Table 3) were between 0.858 and 0.950 (*p*<0.001). The use of the categorical data yielded less agreement among the experts. Nevertheless, the agreement increased substantially in the extremes, *experienced* (kappa = 0.650, *p*<0.001) versus *inexperienced* (kappa = 0.365, *p*<0.001). The uncertainty is marked in the intermediary categories—*moderately experienced* and *less experienced*. Hence, we believe the model worked satisfactorily in agreeing with experts as far as evaluating whether a runner was experienced or not.

The levels of experience were better discriminated when the experts were asked to use a continuous scale (analogic visual scale from 0 to 10). Faced with deciding among few options, experts judged more in accordance when there were more degrees of freedom to choose. In any case, evaluating experience and the experience itself entail processes that involve fuzzy sets and warrant models based on fuzzy logic.

Gardner [34] stated that the understanding of expertise demands investigations into the acquisition of expertise and that entail asking: (1) who is involved in a practice long enough to become an expert and why one does so, (2) who continues to be an expert and why, (3) in what ways do those who continue to be experts differ from all others. Gardner [34] pointed out that the most of the research on the expert focused on the third question. Recent studies have continued to ask the third question, which consequently continues to be at the top of the agenda [30]. Although our research was not designed to address these questions, it nevertheless shows that the understanding of how to evaluate experience and its levels touch on the fundamental question of how experience is acquired.

Experience is a condition that will lead to someone to become an expert, although experience is a prerequisite for being an expert, other factors contribute to expertise [35]. Some 40 years ago, Kay [36] remarked that a common strategy in experimental psychology was to investigate the exceptional in skilled performance. This strategy worked well as in that it identified some factors and mechanisms that affected and underlie skilled behavior. However, if we want to understand how this skilled state is achieved and furthermore improved, Kay [36] emphasized that we must pay attention to the ubiquity of skilled performance that allows practically everyone to become minimally competent in interacting with the physical and social environment. Understanding experience addresses the ubiquity of skilled behavior.

The development of a model is a step toward understand how one can run properly with health and psychosocial benefits. At the same time, increasing participation in competitions, even for fun, has yielded an increase in injuries.

Theisen et al, [37] argue that long-term experience in running (years of practice) is not related to the risk of injury. Furthermore, they do point out that regular practice in the last twelve months has a protective effect against running injuries. Indeed, according to our model years of practice, as well quality of practice, which entails training volume and frequency, is important and complementary feature in the determination of experience level. The absence of relationship between running experience and injury pointed out by Theisen et al[37] maybe a result of the use of years of practice as the sole factor to define experience. As the proposed fuzzy model treats experience taking into account more variables than years of practice, we wonder whether there is indeed no relationship between risk of injury and experience, and if different results would be found if the former author had at their disposal our model.

Some studies have remarked the influence that individual features, such as anthropometric profile, age and gender, might have on kinematic running patterns [38]. In further studies, it would be very promising to verify how experience influences the biomechanical adaptations in running, because there are some evidences that experience in a given motor skill will have a great effect on the technical strategies adopted by the athlete while running.

Regarding on the classical literature on expert performance one would say that running experience would be equated to practice time, and the longer the practice the better as the ten years of practice rule would claim. However, the fuzzy rule-based model points out that running experience depends not only on practice time but also on the quality of practice (training volume and frequency) and number of competitions. Following the fuzzy model, a runner can become well experienced in less than ten years depending upon factors that are related to the quality of experience rather than only on the quantity.

The fuzzy rule-based model can deal with the uncertainty inherent in the judgments on the runner’s experience. It was developed by a combination of variables—quality of training (training volume and frequency) and years of practice/number of races–whose balance varies though with similar results as far as experienced is concerned. It is difficult to have a black and white rule for this as is expressed by the expert’s judgments (running coaches). The grey areas in which their judgment lied was captured by the fuzzy rule-based model developed here making it reliable to evaluate the experience of any new non-elite runner.

The model can also be valid for any runner including elite athletes, because the performance pillars–training volume and frequency, number of races and years of practice–are the same for elite and non-elite runners. However, for this application to elite runners the model would need to be updated by experts particularly in regard to the values of training volume (week mileage) and frequency (number of sessions per week) as they will be certainly differ from those of the non-elite runner. Overall, we are confident on the model’s reliability and validity and it can be a robust system for classifying running experience, with sound applications in sports sciences and human movement studies, sports training, and clinical settings.
